# A Dictionary of Dental Science, Biography, Bibliography and Medical Terminology

**Published:** 1849-04

**Authors:** 


### Bibliographical Notices.

*A Dictionary of Dental Science, Biography, Bibliography and Medical Terminology, by* Chapin A. Harris, M. D., D. D. S. Philadelphia: Lindsay & Blakiston, 1849, pp. 780, royal octavo.

A want which has been long and much felt in the dental profession is now met in the fullest manner. Not only are all medical and surgical terms explained with concise clearness, but we have also a collection of valuable knowledge in all that relates to dental science, which we can find in no single work elsewhere. This dictionary will, among physicians and surgeons, successfully rival our best standard medical dictionaries; whilst among dentists it cannot fail to command a most unquestionable and decided preference, embodying, as it does, all that the others can teach, and much more on which they are silent.

The work has been prepared with much care, at a great expense of labor and time, time which, to the dentist of eminence, is emphatically money. Professional occupation is far more profitable, and less wearing upon the mind, than book making; we feel assured that this dictionary was conceived and completed in the unselfish desire to aid and advance dental science; to record the names and works of those who have raised it to its present position; to perfect the terminology, and the as yet incomplete list of technicalities peculiar to our art; to afford, in a condensed form, that knowledge, so far as it is to be had from books, requisite to the dentist. While others have been *wishing* for some such work as the present one, have felt the want but made no effort to supply it, Professor Harris, with his characteristic promptness, and devotion to his art, and his "*facta non verba*" motto, has done what others talked of, and endeavored to meet the demand.

How successful this effort, the profession will judge, most eminently so, we should say, if our own opinion were asked. We have no intention to enter into a labored eulogy of the work; perhaps we should thus imply some doubt of its reception by the public; we have none. It cannot fail to be well received, and to meet with a constantly increasing demand. The library of the American lawyer, will be no less complete without the commentaries of Kent, than will that of the dental surgeon be without this dictionary. In a just appreciation of his labor by his professional brethren, Dr. Harris looks for the reward of his toil, and we confidently predict that he will find it.

We have to regret that the biographical department is not more complete. This has arisen, we believe, from no want of effort on the part of the author, but from the failure of those applied to, for the requisite information to answer his queries with sufficient promptness and detail. We trust that the obligations which the present are under to the past, will be better felt in our profession, that due tribute will be rendered to those who have aided in its advancement, and that in subsequent editions, the author may have no reason to complain of the "difficulty of procuring accurate reliable information," of its "scanty and indefinite" nature, when obtained.

A word in conclusion, as to the style of the "getting up" of the work. A dictionary, of all works, requires a clear type, good paper and secure binding, all this we find here in an eminent degree, highly creditable to printer, publisher and author. In all of Professor Harris' works, we notice this attention to appearance and it pleases us. An author who writes what is instructive, should not be deterred from presenting his labors in a pleasing form, because, forsooth, some book makers supply by appearance what is lacking in substance. As well might the scholar or the sage find an apology for an ungentlemanly and slovenly attire, in the too frequent folly and shallowness of the exquisite. This attention to externals never injures an author, and is due from him to the reader. P. H. A.

